# Influence of hematoxylin and eosin staining on linear birefringence measurement of fibrous tissue structures in polarization microscopy

**DOI:** 10.1117/1.JBO.28.10.102909

**Published:** 2023-09-30

**Authors:** Liangyu Deng, Chunyan Chen, Wenxi Yu, Conghui Shao, Zan Shen, Yonggang Wang, Chao He, Hongtao Li, Zhiyan Liu, Honghui He, Hui Ma

**Affiliations:** a Tsinghua University, Institute of Biopharmaceutical and Health Engineering, Tsinghua Shenzhen International Graduate School, Guangdong Research Center of Polarization Imaging and Measurement Engineering Technology, Shenzhen Key Laboratory for Minimal Invasive Medical Technologies, Shenzhen, China; b Shanghai Jiao Tong University School of Medicine, Shanghai Sixth People’s Hospital, Department of Pathology, Shanghai, China; c Shanghai Jiao Tong University School of Medicine, Shanghai Sixth People’s Hospital, Department of Oncology, Shanghai, China; d Tsinghua University, Department of Physics, Beijing, China; e University of Oxford, Department of Engineering Science, Oxford, United Kingdom

**Keywords:** polarimetry, polarization microscopy, H&E staining, birefringence, pathology

## Abstract

**Significance:**

For microscopic polarization imaging of tissue slices, two types of samples are often prepared: one unstained tissue section for polarization imaging to avoid possible influence from staining dyes quantitatively and one hematoxylin-eosin (H&E) stained adjacent tissue section for histological diagnosis and structural feature identification. However, this sample preparation strategy requires high-quality adjacent tissue sections, and labeling the structural features on unstained tissue sections is impossible. With the fast development of data driven-based polarimetric analysis, which requires a large amount of pixel labeled images, a possible method is to directly use H&E stained slices, which are standard samples archived in clinical hospitals for polarization measurement.

**Aim:**

We aim to study the influence of hematoxylin and eosin staining on the linear birefringence measurement of fibrous tissue structures.

**Approach:**

We examine the linear birefringence properties of four pieces of adjacent bone tissue slices with abundant collagen fibers that are unstained, H&E stained, hematoxylin (H) stained, and eosin (E) stained. After obtaining the spatial maps of linear retardance values for the four tissue samples, we carry out a comparative study using a frequency distribution histogram and similarity analysis based on the Bhattacharyya coefficient to investigate how H&E staining affects the linear birefringence measurement of bone tissues.

**Results:**

Linear retardance increased after H&E, H, or E staining (41.7%, 40.8%, and 72.5% increase, respectively). However, there is no significant change in the imaging contrast of linear retardance in bone tissues.

**Conclusions:**

The linear retardance values induced by birefringent collagen fibers can be enhanced after H&E, H, or E staining. However, the structural imaging contrasts based on linear retardance did not change significantly or the staining did not generate linear birefringence on the sample area without collagen. Therefore, it can be acceptable to prepare H&E stained slices for clinical applications of polarimetry based on such a mapping relationship.

## 1 Introduction

As a vectorial property, polarization encodes high-dimensional information of light.^1^^–^^3^ Polarimetry can provide abundant optical and structural information of tissue; thus it has shown great potential in biomedical and clinical applications.^4^^–^^10^ After interacting with tissue, the polarization state of light can be altered by different structures.^11^ By measuring the polarization state changes of output light from tissue, several properties, such as birefringence, dichroism, and depolarization, can be quantified.^12^ For microscopic polarization imaging of thin tissue slices, linear birefringence is a valuable and prevalently used metric for anisotropic structures characterization.^13^^–^^15^ For instance, Wood et al.^16^ demonstrated that linear birefringence can be adopted for *in vivo* tissue fiber characterization. Furthermore, Pierangelo et al.^17^ showed that the reducing of linear birefringence resulting from the breaking down of well-ordered fibrous structures can be used for cervical cancer identification.

For polarization microscopy, two types of samples are often prepared: one unstained tissue section for polarization imaging and one adjacent stained tissue section for histological diagnosis and structural feature identification.^18^^–^^20^ Currently, hematoxylin and eosin (H&E) staining is the most popular choice and the cornerstone of anatomical pathological diagnosis.^21^ This convenient and cost-effective dye combination is capable of revealing remarkable cellular details, to the extent that the ultrastructural features can be deduced.^22^ When measuring the linear birefringence property of tissue slices, unstained samples are often used to avoid possible influence from staining dyes quantitatively.^23^^,^^24^ However, this sample preparation strategy brings several other problems. First, the requirement for the preparation of high-quality adjacent tissue sections burdens the technicians. Second, labeling structural features on unstained tissue sections is impossible, and the distortions or rotations between adjacent slices make comparisons at the pixel level impractical. As the fast development of data driven-based polarimetric analysis, which requires a large amount of pixel labeled images, a possible method is to directly use H&E stained slices, which are standard samples archived in clinical hospitals for polarization measurement. There are very few studies that compare the optical properties of unstained and stained tissue samples.^25^^,^^26^ Although the assessment of polarized images of unstained and H&E stained tissues has been conducted previously, a quantitative study on the influence of hematoxylin staining, eosin staining, and H&E staining on linear birefringence measurement of fibrous tissue structures in polarization microscopy is still missing and necessary.

Here we examine the linear birefringence properties of four pieces of adjacent bone tissue slices with abundant collagen fibers, which are unstained, H&E stained, hematoxylin (H) stained, and eosin (E) stained. After obtaining the spatial maps of linear retardance values for the four tissue samples, we carry out a comparative study using a frequency distribution histogram (FDH) and similarity analysis based on the Bhattacharyya coefficient (BC) to investigate how H&E staining affects the linear birefringence measurement of bone tissues. The results reveal no significant change in the linear retardance imaging contrast induced by fibrous structures before and after H&E staining, which cannot generate linear birefringence without collagen fibers. It can be acceptable to directly prepare H&E stained slices to obtain normalized linear retardance images.

## 2 Materials and Methods

### 2.1 Polarimetry Imaging System and Analysis

**Fig. 1:**
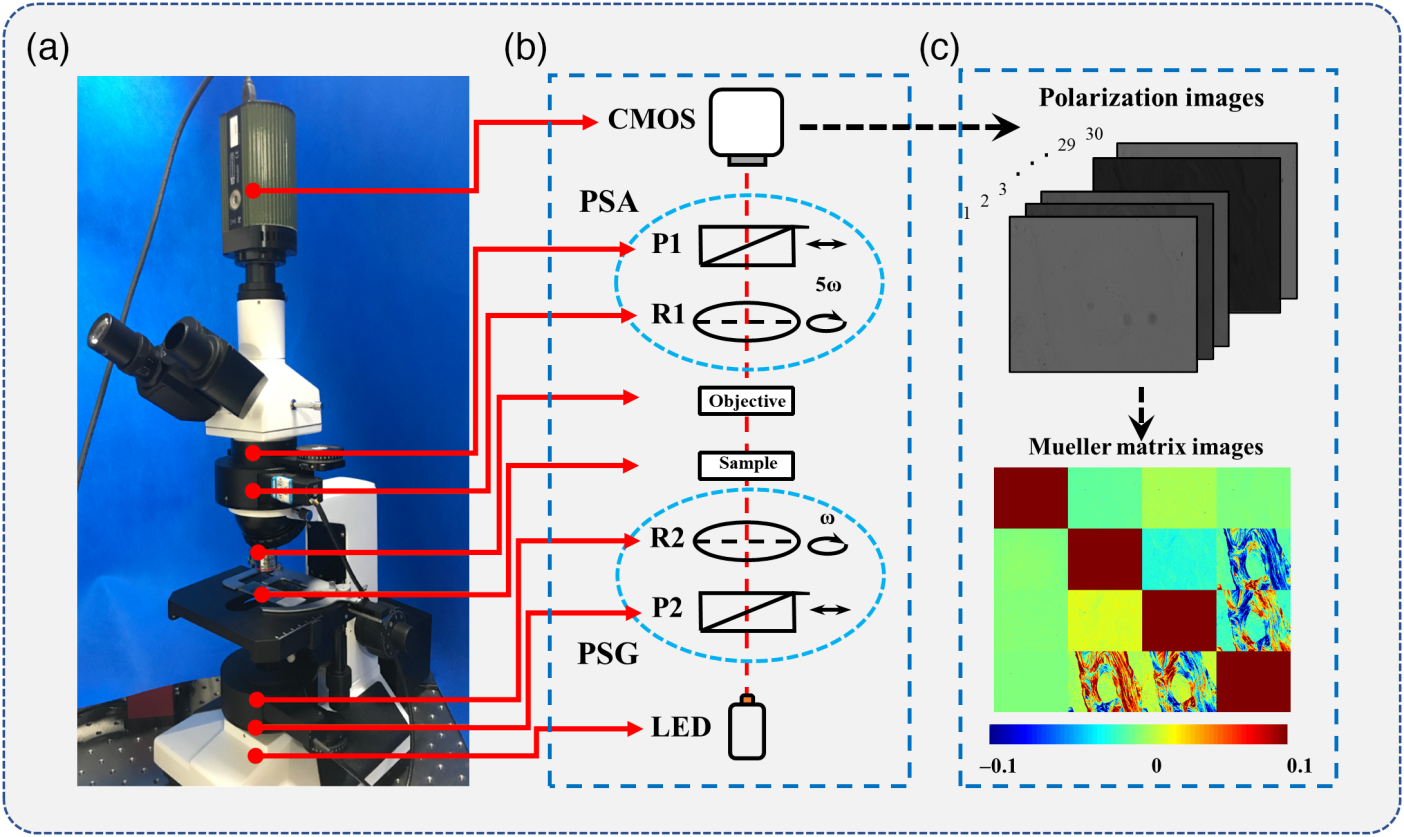
Schematics of (a) transmission MM microscope; (b) the diagram of the device; and (c) flowchart of the MM calculation using the 30 intensity images.

A transmission Mueller matrix (MM) microscope^27^ based on dual-rotating retarders^28^ is employed in this study. As shown in Fig. 1, the illuminating beam from an LED (XLamp XP-E, 633 nm, 3.5 W, Δλ=20  nm, Cree Inc., United States) passes through the polarization state generator module consisting of a fixed linear polarizer (P2, extinction ratio 1000:1, Daheng Optics, China) and a rotatable quarter-wave plate (R2, Daheng Optics, China) to control the input state of polarization (SOP). Here 30 different elliptical SOPs are generated by rotating the R2. After interacting with the sample, the transmitted light passes through the objective lens (4×/0.1 NA, UPlanSApo, Olympus, Japan) and then is detected by the polarization state analyzer (PSA) module consisting of a rotatable quarter-wave plate (R1, Daheng Optics, China) and a fixed linear polarizer (P1, extinction ratio 1000:1, Daheng Optics, China). Finally, the light carrying the polarization related information of the sample is received by a CMOS camera (MV-CA016-10UM, 12-bit, Hikvision, China) and stored as a 1080×1440  pixels intensity image (3.45  μm×3.45  μm pixel size).

During each measurement, the P1 and P2 are fixed in the horizontal direction, and both the R1 and R2 are synchronously rotated at different angular speeds ($\omega_{R1}:\omega_{R2}=5:1$) to achieve 30 different SOP modulations. Because the detected light intensity is a periodic signal, the MM is calculated based on the Fourier analysis using the coefficients $\alpha_{n}$ and $\beta_{n}$ as shown in the following equation:

$$I=\alpha_{0}+\overset{12}{\underset{n=1}{\sum }}(\alpha_{n}\;\cos\;2n\theta +\beta_{n}\;\sin\;2n\theta ),$$ (1)

where I is the light intensity collected by the camera, the Fourier coefficients $\alpha_{n}$ and $\beta_{n}$ are the functions of the 16 MM elements, and θ is the rotation angle of the quarter-wave plate R1. Before measurement, the MM microscope was calibrated using standard samples to ensure that the maximum error of the individual element is within 1%. The detailed calibration procedure can be found in our previous work.^29^

For pathological tissue slices with several microns thickness, their depolarization and diattenuation properties are often limited.^30^ However, the birefringence induced by anisotropic structures in tissue sections can provide abundant useful information for label-free diagnosis.^31^ When propagating through fibrous structures with linear birefringence, polarized light undergoes a linear phase retardance δ, which is quantified as

$$\delta =\frac{2\pi \Delta nd}{\lambda },$$ (2)

where Δn is the refractive index difference, d is the optical path length, and λ is the wavelength of light. Thus for standard pathological tissue slices with relatively constant thickness d, the linear retardance δ is proportional to Δn and is used as a quantitative measure of abnormal tissue areas with different fibrous microstructures. Here to obtain the value of linear retardance δ, we adopt the Mueller matrix polar decomposition (MMPD) method prevalently used in tissue polarimetry.^12^ The MMPD method decomposes a MM into three main properties, namely diattenuation (D), retardation (R), and depolarization (Δ), as shown in the following equation:

$$M=M_{\Delta }M_{R}M_{D},$$ (3)

where $M_{D}$, $M_{R}$, and $M_{\Delta }$ are the diattenuation, retardation, and depolarization submatrices, respectively. Then the retardation matrix $M_{R}$ is further decomposed to obtain the magnitude of linear retardance δ as shown in the following equation:^12^

$$\delta =\cos^{-1}(\sqrt{{\left(M_{R}(2,2)+M_{R}(3,3)\right)}^{2}+{\left(M_{R}(3,2)-M_{R}(2,3)\right)}^{2}}-1),$$ (4)

where $M_{R}(i,j)$ are the elements of $M_{R}$.

### 2.2 Tissue Samples

**Fig. 2:**
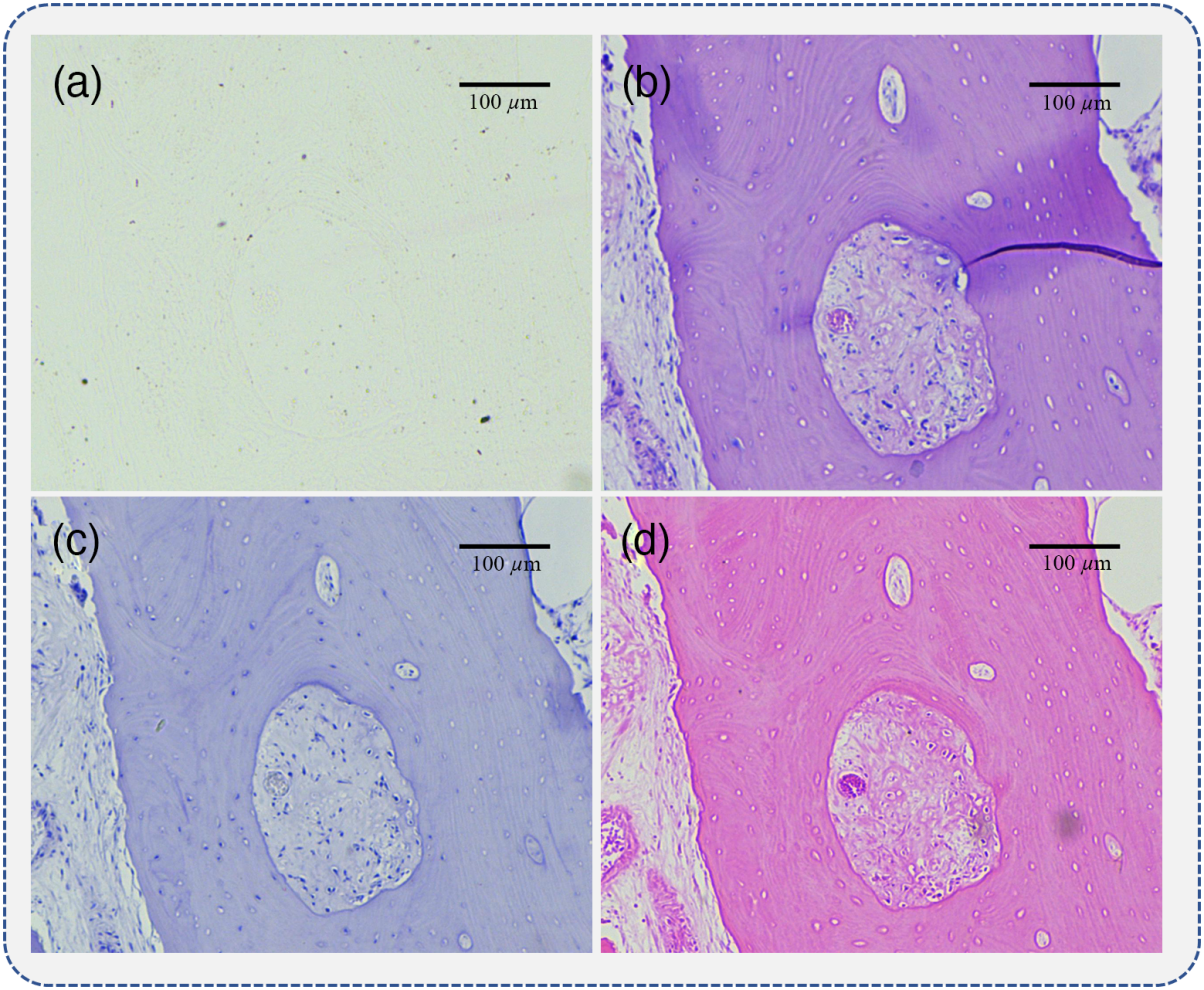
Bright field images of four adjacent bone tissue slices: (a) the dewaxed unstained slice; (b) the H&E stained slice; (c) the H stained slice; and (d) the E stained slice.

For quantitative evaluation of the influence of H&E staining on the linear retardance measurement of tissue slices, in this study, the human bone tissue samples with strong birefringence were used.^32^ Specifically, 20 human bone tissue samples were included in this study, with 10 samples representing normal and 10 samples representing abnormal bone pathological conditions. For comparison, four adjacent 4-μm thick slices of each bone tissue dehydrated and embedded in paraffin were prepared to produce unstained, H&E stained, hematoxylin (H) stained, and eosin (E) stained versions by an experienced pathologist from the Shanghai Sixth People’s Hospital. The bright field images of the four adjacent bone tissue slices are shown in Fig. 2. It can be observed that the dewaxed unstained slice image shown in Fig. 2(a) only provides the contour information. However, in the stained slices images shown in Figs. 2(b)–2(d), the nuclei and cytoplasm are highlighted as blue and pink colors by H and E staining, respectively. For the unpolarized images of the four adjacent slices shown in Fig. 2, the location and distribution of bone tissue structures are roughly the same, which permits direct comparisons between the corresponding polarimetric imaging results of the four slices to analyze how H&E staining affects the linear retardance measurement. This work was approved by the Ethics Committee of Shanghai Sixth People’s Hospital Affiliated to Shanghai Jiao Tong University School of Medicine.

## 3 Results and Discussion

**Fig. 3:**
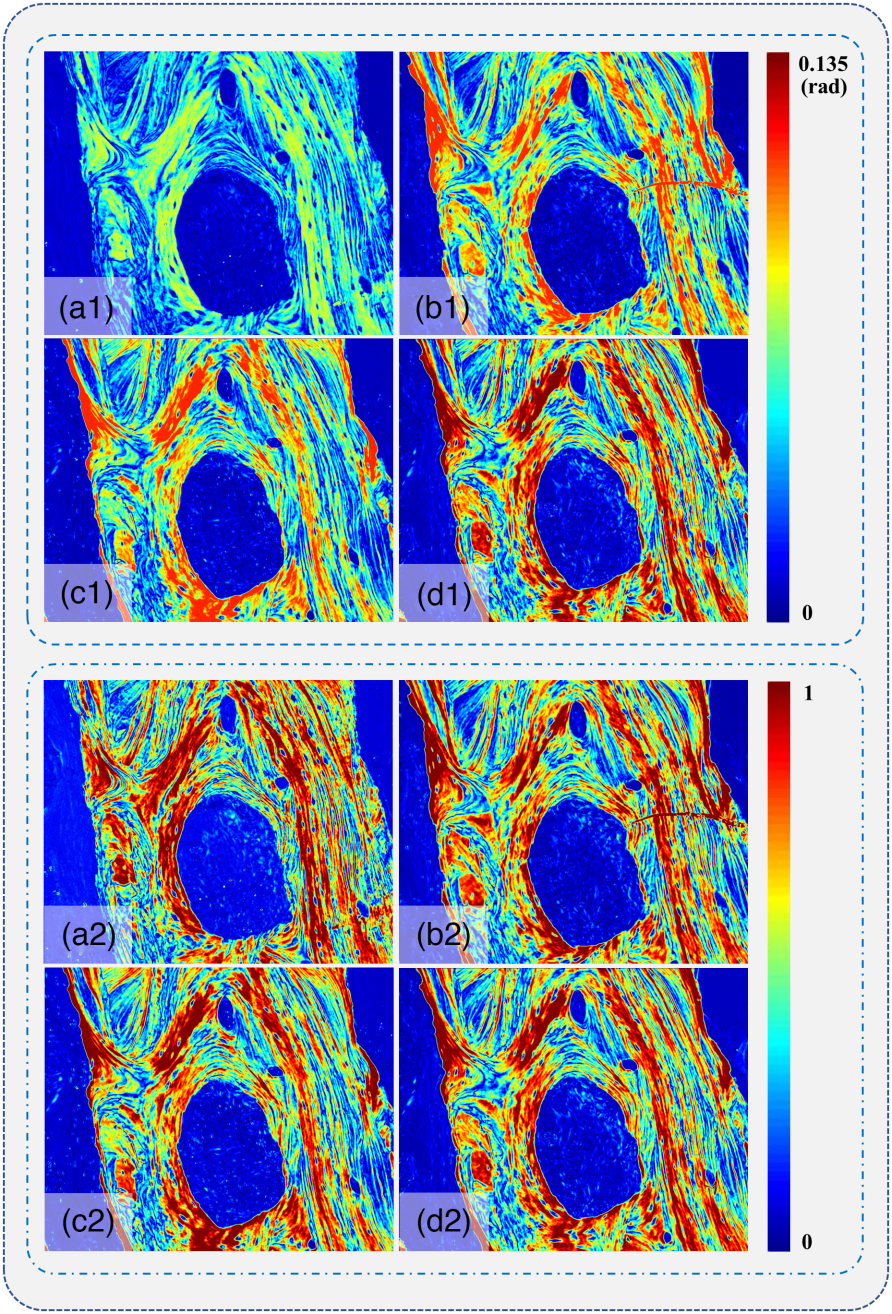
Linear retardance parameter δ images of the bone tissue slices: (a1)–(d1) the linear retardance images for the unstained, H&E stained, H stained, and E stained slices, respectively, and (a2)–(d2) the corresponding normalized images.

The linear retardance δ images for the bone tissue slices are shown in Fig. 3. Obviously, bone tissue structures have prominent linear birefringence effect to produce significant contrast in parameter δ images. Previous studies have demonstrated that the strong birefringence effect mainly comes from collagen fibers, with density and orientation that can be quantitatively evaluated using linear retardance parameters.^33^ In this study, we choose the bone tissue specimens with abundant fibrous structures as the samples for comparative studies on the influence of H&E staining on linear birefringence measurements.

It can be observed from the un-normalized linear retardance images shown in Figs. 3(a1)–3(d1) that the distributions of the birefringent structures are basically the same, regardless of the pixel values differences. This indicates that the hematoxylin, eosin, and H&E staining cannot produce linear birefringence to the non-birefringent tissue areas. In other words, it suggests that the dye itself cannot generate significant changes in Δn of the tissue without linear birefringence effect, according to Eq. (2).

Meanwhile, Fig. 3 also shows that the linear birefringence of the fibrous structures may be enhanced by the attached dye. For a demonstration, we calculated the mean values of linear retardance δ for the bone tissue slices shown in Figs. 3(a1)–3(d1). Here a pixel-by-pixel comparison of the values is impractical due to the displacement and slight rotation of different slices during preparation. The mean linear retardance δ values of the unstained [Fig. 3(a1)], H&E stained [Fig. 3(b1)], H stained [Fig. 3(c1)], and E stained [Fig. 3(d1)] slices are 0.0316, 0.0448, 0.0445, and 0.0545 rad, respectively. In other words, compared with the unstained bone tissue slice, the E and H staining enhanced bone tissue samples’ δ values by 72.5% and 40.8%, respectively. Hence, the mean linear retardance δ value of the H&E stained slice with both the hematoxylin and eosin dyes increased by 41.7%. This indicates that the combination of dye molecules and birefringence structures, which are mainly the collagen fibers in bone tissues, increases the linear birefringence property. Similarly, previous studies have shown that the dye of Sirius Red can also significantly enhance the birefringence of collagen fibers,^34^^–^^36^ the mechanism of which is the elongated dye molecules attached to the collagen fibers with their long axes parallel to the collagen orientation. This parallel relationship between dye molecules and collagen fibers results in an enhanced linear birefringence. Here a possible reason for the results shown in Fig. 3 is that both the hematoxylin and eosin molecules can be attached to the collagen fiber in a similar parallel way as that of the Sirius Red molecules, leading to the enhancement of linear birefringence.

**Fig. 4:**
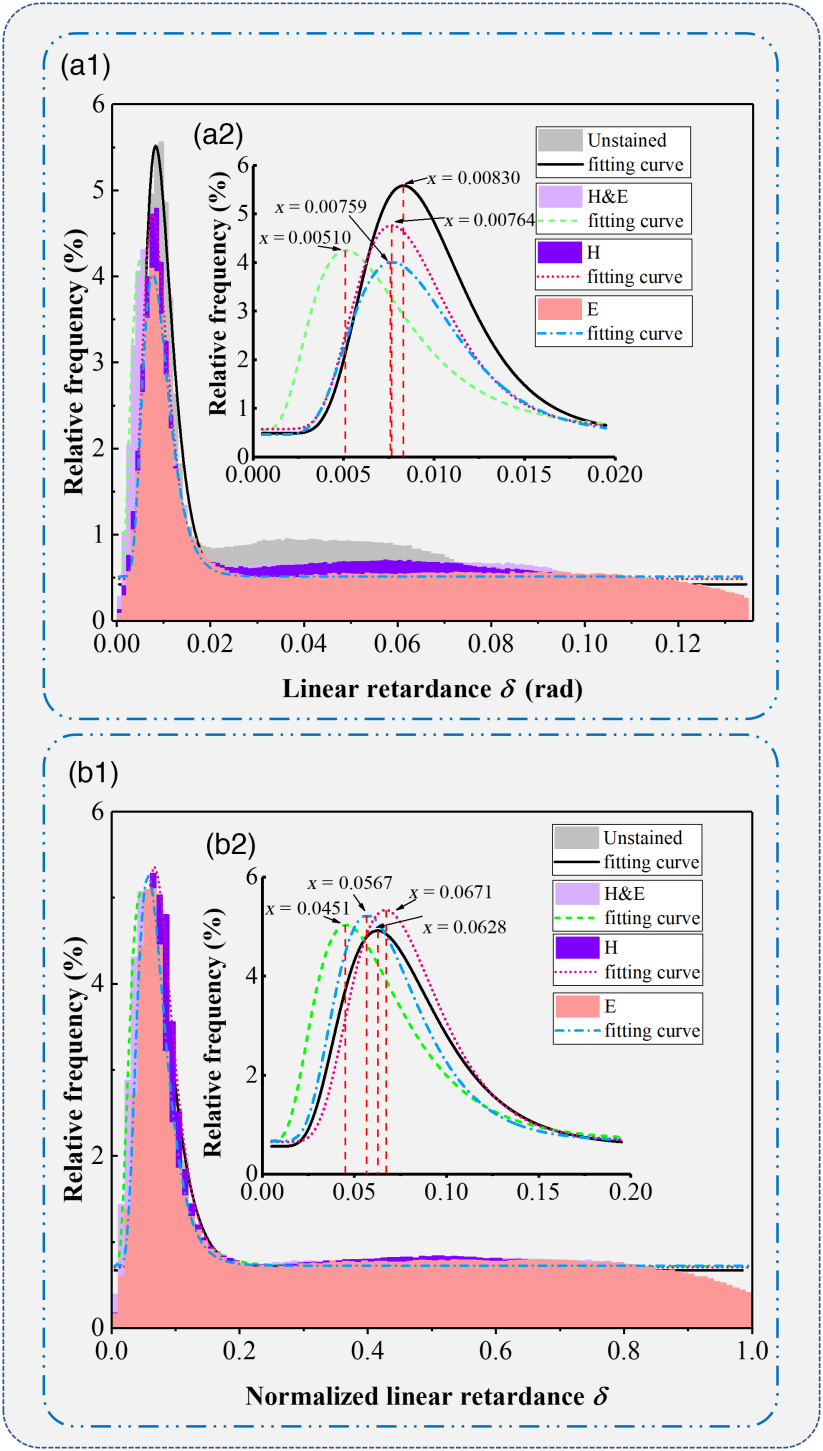
Frequency distribution histogram curves of the linear retardance images of bone tissue slices: (a1), (a2) the frequency distribution histograms of the original linear retardance images, in which (a2) is the subgraph of (a1) counting in [0, 0.02]; (b1), (b2) the frequency distribution histograms of the normalized linear retardance images, in which (b2) is the subgraph of (b1) counting in [0, 0.2].

In addition to the global enhancement of linear birefringence values, it can also be noticed from Figs. 3(a1)–3(d1) that the imaging contrasts between collagen fibers and other areas are kept in different stained bone tissue samples. For a demonstration, Figs. 3(a1)–3(d1) images are normalized to generate images shown in Figs. 3(a2)–3(d2). We can see that the normalized images of different stained tissue samples maintain very similar imaging contrasts, which reflect the density and distribution of collagen fibers. To evaluate such kind of imaging contrast consistency in detail, we performed FDH analysis on the four linear retardance images before and after normalization, as shown in Fig. 4. The FDHs of the four un-normalized images in Fig. 4(a) all represent the same fluctuating distributions of a sharp decline after reaching the peak value and then gently maintain a base value. Also it can be observed that the frequencies of linear retardance values are concentrated around different peaks for different stained bone tissues. The maximum deviation of the linear retardance value at the peak for Fig. 4(a) is 2.56%. After the normalization, the linear retardance images of different stained bone tissue slices tend to have more similar FDH distributions as shown in Fig. 4(b), with the maximum deviation of the linear retardance value at the peak being 2.2%. It confirms that the linear birefringence of the bone tissue slices was enhanced after H&E staining. However, the structural imaging contrasts based on linear retardance did not change significantly or the staining cannot generate linear birefringence on the sample area without collagen.

For a more detailed quantitative comparison, we then divided each normalized linear retardance image shown in Figs. 3(a2)–3(d2) into nine subimages of the same size and performed similarity analysis on the corresponding subimages pair to judge whether they still maintain the imaging contrast consistency after staining. The original image of 1080×1440  pixels was linearly divided into nine equal-sized subimages of 360×480  pixels. Here we use the BC as shown in Eq. (5) for the similarity evaluation:

$$B(H_{1},H_{2})=\sqrt{1-\underset{i}{\sum }\frac{\sqrt{H_{1}(i)\cdot H_{2}(i)}}{\sqrt{\underset{i}{\sum }H_{1}(i)}\cdot \sqrt{\underset{i}{\sum }H_{2}(i)}}},$$ (5)

where $H_{1}(i)$ and $H_{2}(i)$ are the two frequency distribution functions of corresponding subimages pair and i represents the median value of each interval after the linear retardance value range is divided into 100 equal intervals.

The BC is a popular statistical method for measuring the similarity of two normalized distributions.^37^ It has been prevalently used in various fields ranging from classical statistics to artificial intelligence because of its simplicity and robustness to small outliers.^38^ Previous study indicated that a perfect overlap between two distributions yields a BC value of exactly 1. Also a BC value around 0.8 means that the two distributions still maintain the corresponding distribution peaks and valleys and the morphological deviation between the two is not significant.^39^ As we can see from Table 1, the average BC values between the unstained and different stained bone tissues are 0.8244, 0.8360, and 0.7849, respectively, indicating that the linear retardance distribution had a large overlap after the H&E, H, or E staining. Here we focus on the H&E stained subimages considering their broad pathological application. Except for subimages 3 and 6, the BC values calculated between the unstained and H&E stained slices are >0.8, which means that the pixels value distribution after H&E staining remains relatively consistent. The BC value lower than 0.8 in area 6 may be due to the artifacts induced by the occurrence of folds in the tissue section preparation process.

**Table 1:** Similarity analysis results using the BC on the corresponding normalized subimage pair.

		H&E	H	E
Unstained	Area 1	0.8506	0.8497	0.8950
	Area 2	0.8714	0.8426	0.7528
	Area 3	0.6718	0.8001	0.7000
	Area 4	0.8065	0.8522	0.8474
	Area 5	0.8952	0.8200	0.8536
	Area 6	0.7392	0.8300	0.7022
	Area 7	0.8131	0.8994	0.8096
	Area 8	0.8673	0.8953	0.7989
	Area 9	0.9049	0.7343	0.7049
	Average	0.8244	0.8360	0.7849

Overall, a significant similarity exists in four different versions of the same tissue sample. As for the regions with higher similarity, they mainly reflect the birefringent pathological features, such as the fibrous bone structures, with linear retardance imaging contrast that is reserved in the measurement of stained slices. As for the regions with relatively low similarity, this may be due to the artifacts induced by the sample preparation process rather than specific pathological features.

In addition, we computed the BC among the four variants within each sample in a broader sample perspective, utilizing the identical calculation method as described earlier. As illustrated in Table 2, the coefficients represent the average BC values across 10 normal bone tissue samples, yielding an overall average similarity of 0.8081, 0.8101, and 0.8300 for H&E, H, and E stained samples compared with the unstained sample, respectively. Moreover, the global similarities for the abnormal bone tissue samples, as depicted in Table 3, reached 0.7835, 0.8004, and 0.7979 for H&E, H, and E staining, respectively. The statistical analysis results based on the expanded sample size indicate that H&E, H, or E staining has a minor impact on the overarching structural contrast in both normal and abnormal bone tissue samples.

**Table 2:** Similarity analysis results using the BC on the corresponding normalized image pair of normal human bone tissue samples.

	Normal samples	H&E	H	E
Unstained	Sample 1	0.7849	0.8244	0.8360
	Sample 2	0.8687	0.8792	0.8700
	Sample 3	0.7597	0.7575	0.7552
	Sample 4	0.8212	0.8265	0.8367
	Sample 5	0.7639	0.7682	0.8270
	Sample 6	0.8375	0.8327	0.8580
	Sample 7	0.8136	0.8129	0.8417
	Sample 8	0.8217	0.8146	0.8424
	Sample 9	0.7982	0.7959	0.8240
	Sample 10	0.8118	0.7890	0.8088
	Average	0.8081	0.8101	0.8300

**Table 3:** Similarity analysis results using the BC on the corresponding normalized image pair of abnormal human bone tissue samples.

	Abnormal samples	H&E	H	E
Unstained	Sample 1	0.8403	0.8247	0.8232
	Sample 2	0.7462	0.7599	0.7603
	Sample 3	0.7551	0.7661	0.7518
	Sample 4	0.7954	0.8033	0.7900
	Sample 5	0.7658	0.7612	0.7596
	Sample 6	0.8066	0.8285	0.8192
	Sample 7	0.7213	0.7683	0.7998
	Sample 8	0.8319	0.8387	0.8336
	Sample 9	0.8186	0.7766	0.7922
	Sample 10	0.7541	0.8767	0.8497
	Average	0.7835	0.8004	0.7979

**Fig. 5:**
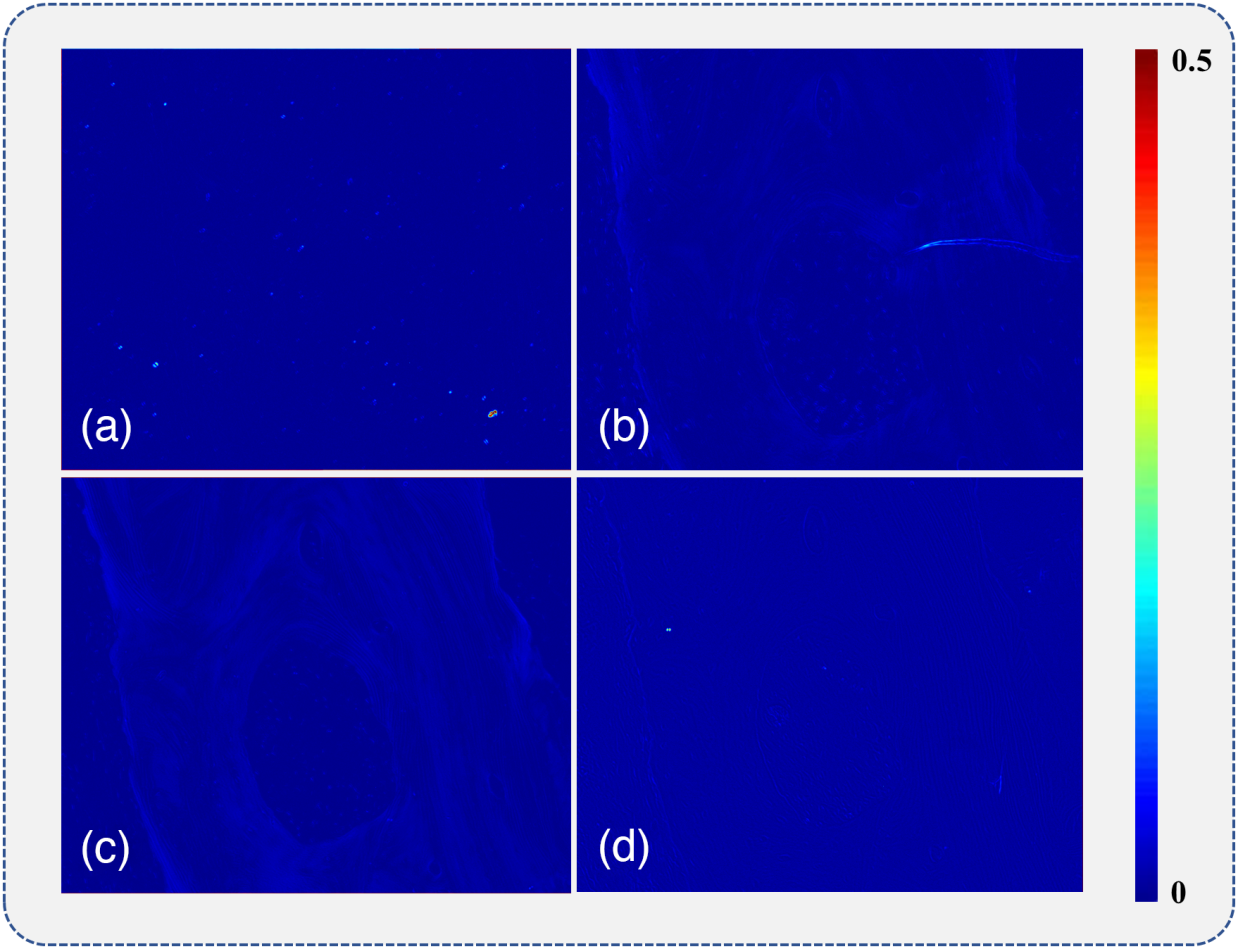
Diattenuation D images of (a) unstained, (b) H&E stained, (c) H stained, and (d) E stained bone tissue slices.

To show the linear dichroism of the samples, we also calculated the MMPD diattenuation parameter D according to the following equation:

$$D=\sqrt{M_{12}^{2}+M_{13}^{2}+M_{14}^{2}},$$ (6)

where $M_{ij}$ (i,j=1, 2, 3, 4) represents the corresponding MM element. The diattenuation parameter D images are shown in Fig. 5, where the average D values are 0.0060, 0.0084, 0.0082, and 0.0069 for the unstained, H&E stained, H stained, and E stained slices, respectively. The calculation also indicates that the maximal D values are 0.0181, 0.0288, 0.0274, and 0.0146 for the unstained, H&E stained, H stained, and E stained slices, respectively. According to the absorption spectra of the dyes in solution,^40^^–^^43^ the spectra range of hemalum complexes, which are active ingredients in the hematoxylin staining process, is from ∼400 to 700 nm, producing a maximal peak at 566 nm, whereas the spectra range of eosin molecules is from ∼440 to 550 nm with the maximal peak at 517 nm. Thus when the 633 nm LED was used as the light source of the MM microscope in this study, the H&E and H stained tissue slices exhibited a slightly linear dichroism enhancement (∼0.002 for the average value and 0.01 for the maximal value) as shown in Figs. 5(b) and 5(c). It may be due to those dye molecules, especially hemalum complexes attaching to the collagen fibers. However, the results shown in Fig. 5 also confirm that both the intrinsic linear dichroism effects of the samples and the enhancement of diattenuation induced by staining are limited. It can produce very small additional contrast to the linear retardance images.

In summary, both the FDH and BC analysis results reveal no significant change in the image contrast of linear retardance induced by the fibrous structure after H&E staining, which cannot generate linear birefringence without collagen fibers. Such a mapping relationship can be used for clinical application of polarimetry; for instance, for pathological tissue samples with significantly altered fibrous structure, the measurement of the linear retardance in the H&E stained slice can reflect the information of birefringent fibrous structure in the corresponding unstained slice. It may be acceptable to directly prepare H&E stained slices to obtain normalized linear retardance images for reducing sample preparation workload in pathological tissue diagnosis, which mainly focuses on the imaging contrast between different tissue structures rather than to obtain adjacent tissue slices for measuring and staining^44^^–^^46^ or measure unstained slices first and then counterstaining them.^47^ For instance, the characteristic polarization imaging contrast produced by linearly birefringent collagen orientation is applicable in detecting common bone diseases such as fragility fractures and osteoporosis.^48^ However, it should be noted that the feasibility of directly utilizing H&E stained slices for polarization measurements may vary depending on the specific objectives, particularly if the focus is on absolute linear birefringence values, which can be changed by H&E staining. Meanwhile, polarization measurement data based on H&E stained slices can be more conveniently obtained, analyzed, and labeled because the pathological slices archived in the hospital are mainly H&E stained slices for retrospective research instead of unstained slices.^49^

## 4 Conclusion

In this study, we quantitatively analyzed the effect of H&E staining on linear birefringence imaging results. We measured four adjacent pieces of bone tissue slices, which were unstained, H&E stained, hematoxylin (H) stained, and eosin (E) stained. The comparison results of mean retardance values showed that the linear retardance values induced by birefringent collagen fibers can be enhanced after H&E, H, or E staining. The increasing magnitudes were 41.7% for H&E staining, 40.8% for H staining, and 72.5% for E staining compared with that of the unstained tissue slice. Furthermore, the FDH and BC analysis results confirmed that the linear birefringence of the bone tissue slices was enhanced after H&E staining. However, the structural imaging contrasts based on linear retardance did not change significantly, or the staining did not generate linear birefringence on the sample area without collagen. Therefore, it can be acceptable to prepare H&E stained slices for some pathological polarized imaging situations based on such a mapping relationship, focusing on obtaining the imaging contrast between different tissue structures. With the fast development of data-driven based polarimetric analysis, which requires a large amount of pixel labeled images, an effective method is to directly use H&E stained slices, which are standard samples archived in clinical hospitals for polarization measurements.

## Code, Data, and Materials Availability

Data underlying the results presented in this paper are not publicly available at this time but may be obtained from the authors upon reasonable request.
